# Dung Beetles Eat Acorns to Increase Their Ovarian Development and Thermal Tolerance

**DOI:** 10.1371/journal.pone.0010114

**Published:** 2010-04-09

**Authors:** José R. Verdú, José L. Casas, Jorge M. Lobo, Catherine Numa

**Affiliations:** 1 I. U. CIBIO, University of Alicante, Alicante, Spain; 2 Museo Nacional de Ciencias Naturales, CSIC, Madrid, Spain; University of Alabama, United States of America

## Abstract

Animals eat different foods in proportions that yield a more favorable balance of nutrients. Despite known examples of these behaviors across different taxa, their ecological and physiological benefits remain unclear. We identified a surprising dietary shift that confers ecophysiological advantages in a dung beetle species. *Thorectes lusitanicus*, a Mediterranean ecosystem species adapted to eat semi-dry and dry dung (dung-fiber consumers) is also actively attracted to oak acorns, consuming and burying them. Acorn consumption appears to confer potential advantages over beetles that do not eat acorns: acorn-fed beetles showed important improvements in the fat body mass, hemolymph composition, and ovary development. During the reproductive period (October-December) beetles incorporating acorns into their diets should have greatly improved resistance to low-temperature conditions and improved ovarian development. In addition to enhancing the understanding of the relevance of dietary plasticity to the evolutionary biology of dung beetles, these results open the way to a more general understanding of the ecophysiological implications of differential dietary selection on the ecology and biogeography of these insects.

## Introduction

The main reason animals eat is to acquire the mixture of nutrients required to fuel growth, development, and reproduction. A high-quality diet for insects permits the storage of different compounds (e.g., lipids, carbohydrates, or proteins) in the fat body, the most important organ for nutrient metabolism and storage in insects [1]. In fact, the fat body plays a crucial role not only in lipid storage and transport to the hemolymph, but also in lipid biosynthesis from carbohydrates, the biosynthesis of proteins such as antifreeze proteins (AFP) [2], and yolk deposition in oocytes or vitellogenesis [3]. Moreover, the fat body is the major organ for storing glycogen, the precursor of many cryoprotectants (mainly polyhydric alcohols such as glycerol and sorbitol and sugars such as trehalose) [4], [5], [6]. As a measure of cold thermal tolerance, the supercooling point (the temperature at which the insect freezes, SCP) is generally used to assess the cold acclimation and resistance. In cold conditions, freeze-avoiding insects may adopt one or both of these main mechanisms to decrease the SCP: (a) the production of AFPs that inhibit the growth of ice, modify the thermal hysteresis (the difference between the freezing point and the melting point of a solution) and stabilize supercooled hemolymph and other fluids [7], [8], [9], [10], and (b) the adjust concentration of multi-component cryoprotectant compounds that depress the SCP [11].

Nutritional changes can alter thermal tolerance by their influence on the production of antifreeze proteins [12], [13]. Although other dietary needs than the gathering of energy may be critical, the general predictions of the optimal foraging theory is that organisms forage to maximize their net rate of energy intake with the minimum loss of time; the animal increases its fitness by adopting feeding patterns that maximize the intake per unit time of some critical dietary currency, generally energy [14]. Some frequent predictions of this theory are “ that large foods should be preferred to small, patchy foods to spaced-out foods; calorie-rich foods to calorie-poor foods”. However, animals may eat different foods in proportions that yield a more favorable balance of nutrients than from any one of these foods alone, a behavioral strategy called dietary self-selection [14], [15], [16], [17]. Here we show for the first time how the diet diversification of a dung-beetle species to consume acorns allows the improvement of thermal tolerance and reproductive capacity under laboratory conditions. This, in turn may help to explain some ecological and distributional data of this species.

**Figure 1:**
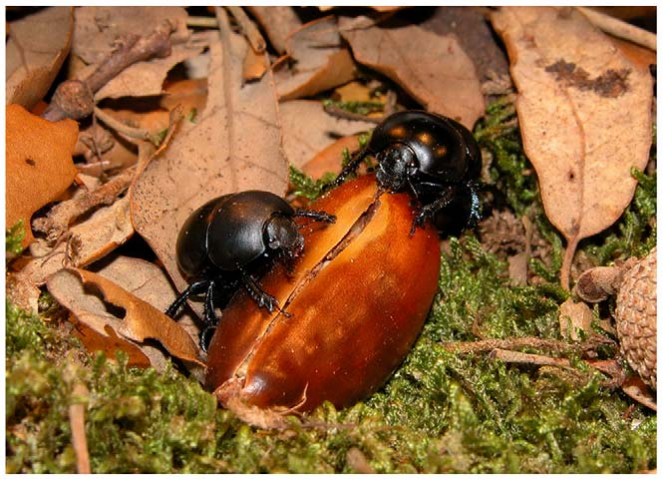
*Thorectes lusitanicus* eating cork-oak acorns. During their reproductive period, *T. lusitanicus* bury and feed on acorns of *Quercus*, with important ecological implications.

*Thorectes lusitanicus* (Scarabaeoidea, Geotrupinae) is an apterous dung beetle species endemic to the southern Iberian Peninsula. Recently, an astonishing and never before observed relationship between this dung beetle (*Thorectes lusitanicus*) and two oak species (*Quercus suber* and *Q. canariensis*) has been described for the Iberian Peninsula [18], [19]. Additionally, this behavior has been also recently described for *Mycotrupes lethroides*, a Geotrupinae dung beetle from Florida [20] as well as for the Iberian species *Thorectes baraudi*, the phylogenetically closest species to *T. lusitanicus* (unpublished data). *Thorectes lusitanicus* acts as a secondary disperser, capable of partially consuming (Fig. 1) dragging and burying a high number of viable acorns of both *Quercus* species during their reproductive period of activity (autumn-winter). Additionally, using olfactometry under laboratory conditions we have shown that specimens of *T. lusitanicus* are significantly attracted by acorns during their reproductive period, even preferring this food resource over the dung necessary to lay their eggs for breeding purposes [19]. During the autumn and winter, acorns are one of the most abundant and nutritious foods in temperate areas where oaks provide the principal forest structure. Nutritionally, acorns contain larger amounts of proteins (18.1%) and lipids (6.1%) [21] than cow dung (5% proteins and 0.4% lipids) [22]. Thus, although the tannins in acorns can cause serious damage to some mice [23], several wild animals, including mammals (such as red deer, wild boars and mice) and birds (such as Eurasian jays and common wood pigeons) consume acorns as a key part of their diet during the autumn and winter. In the case of *T. lusitanicus* the period of acorn production coincides with the reproductive period of this species [24].

In addition to the possible ecological implications for forest regeneration, there is a great interest in understanding the advantages of this dietary shift in dung beetles from dung to acorns, as well as the possible physiological and reproductive implications of this diversification in the trophic niche. In this work, we present experimental evidence that helps to explain the possible benefits of this striking behavior [19]. Given the high content in polyunsaturated fatty acids, triglycerides, and sterols present in the acorns [25], we demonstrate that this diet expansion significantly affects fat body development, hemolymph composition, cold resistance and ovary development under laboratory conditions. Additionally we also include some field data and ecological and natural history evidences supporting the relevance of acorn consumption under natural conditions, thus suggesting that some insects probably have the capacity to enhance their environmental and reproductive capacity by changing their trophic behavior.

## Results

**Figure 2:**
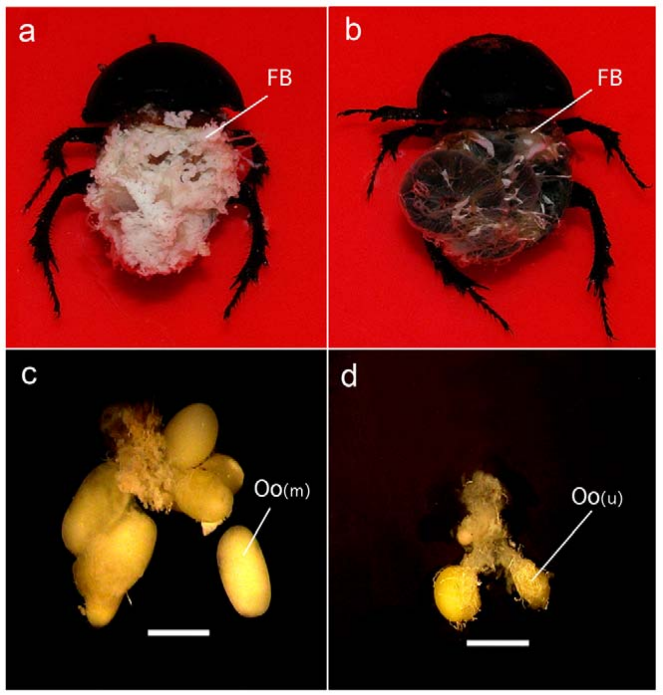
The effect of diet on the fat body and ovary development in *T. lusitanicus*. Diets based on acorns (A and C) and cow dung (B and D). FB: fat body; Oo(m): mature oocyte; Oo(u): undeveloped oocyte.

Beetles fed with acorns developed a fat body 4.6-fold heavier than those fed with cow dung (Table 1). The impressive size reached by this tissue in acorn-fed beetles was even evident to the naked eye (Fig. 2A).

**Table 1:** The effect of food on the fat body mass, hemolymph composition, thermal tolerance and activity of *Thorectes lusitanicus.*

	Food source		
	Acorns	Cow dung	Samples/diet
Fat body (% body mass)	11.26±2.85	2.46±0.85	n = 10
Glycerol (g/L)	0.027±0.009	0.017±0.0002	n = 10
Protein (mg/ml)	129.29±16.36	28.34±4.13	n = 10
SCP (°C)	−13.48±1.15	−8.50±2.17	n = 10
THA (°C)	0.64±0.04	0.41±0.02	n = 5
Palmitic acid (mg/ml)	1.25±0.25	0.39±0.06	n = 3
Palmitoleic acid (mg/ml)	0.12±0.01	0.04±0.04	n = 3
Stearic acid (mg/ml)	0.15±0.02	u.c.	n = 3
Oleic acid (mg/ml)	3.75±0.45	1.21±0.27	n = 3
Linoleic acid (mg/ml)	0.81±0.13	0.40±0.12	n = 3
Linolenic acid (mg/ml)	0.02±0.03	u.c	n = 3
Activity at 20°C (%)	91.3±2.4	43.6±7.9	n = 6
Activity at 10°C (%)	85.5±4.3	17.2±4.9	n = 6

(Mean ± sd). All measurements are statistically significant (P≤0.01) according to a Mann-Whitney non-parametric test. SCP: Supercooling point; THA: Thermal hysteresis; u.c.: undetectable concentrations (≈0).

The hemolymph composition was also extensively altered by the diet, particularly in the fatty acid type and content and in the protein content. All fatty acids found in the hemolymph of cow dung-fed beetles (palmitic, 16:0; palmitoleic, 9-cis 16:1; oleic, 9-cis 18:1; and linoleic acid, 9,12-cis 18:2) increased two to three fold in the acorn-fed beetle hemolymph (Table 1). The acorn diet also determined the presence in hemolymph of stearic (18:0) and linolenic acid (9,12,15-cis 18:3), which were both undetectable under the cow dung diet (Table 1).

Probably, the most outstanding feature of the hemolymph of the acorn-fed beetles was related to the protein content, which was 4.6 times higher than that found in the hemolymph of cow dung-fed beetles (Table 1). Although the type and identity of these proteins are now currently being investigated in our laboratory, a first consequence of this great difference in the protein content derived from the diet was the variation in some biophysical features of the hemolymph (Table 1). We found a reduction in the supercooling point (SCP) from −8.50±2.17°C (mean ± sd) in the hemolymph of cow dung-fed beetles to −13.48±1.15°C of that of the acorn-fed beetles. Besides the modification of the hemolymph SCP, the diet also brought about a change in the thermal hysteresis activity (THA) detected in the hemolymph, which increased from 0.41±0.02°C in the cow dung-fed beetles to 0.64±0.04°C in the acorn-fed beetles. Glycerol was found at similar concentrations in the hemolymph irrespective to the diet (Table 1).

Behavioral data on the activity of *T. lusitanicus* under laboratory conditions showed that those individuals feeding on acorns were significantly more active at the two examined environmental temperatures. Ninety-one percent and 85.5% of individuals feeding on acorns remain active both at 20°C and 10°C, while 43.6% and 17.2% of individuals feeding on cow dung were active at both temperatures, respectively (Table 1).

Finally, beetles fed with acorns also showed a higher development of their ovaries, a higher number of mature oocytes per ovary, and a higher mean weight of oocytes that beetles fed with cow dung. Furthermore, the number of undeveloped ovaries was higher in beetles fed with cow dung (Table 2) than in beetles fed acorns.

**Table 2:** The effect of food on correlates of reproductive success in *Thorectes lusitanicus*.

	Food source	
	Acorns	Cow dung
Ovary weight (% body mass)	3.66±1.03	0.87±0.28
Number of oocytes/ovary	2.10±0.55	0.40±0.22
Mean weight of oocytes (mg)	0.011±0.002	0.003±0.002
Number of undeveloped ovaries	1	7

All measurements are statistically significant (P≤0.01) according to a Mann-Whitney non-parametric test (n = 10 for each diet) (Mean ± sd).

## Discussion

In insects, the main indicator of the nutritional reserve level is the fat body, which appears as a spongy, aggregated mass of cells (trophocytes), whitish in color, and irregularly distributed in the perivisceral space of the thorax and the abdomen. It is known that the fat body is responsible for the synthesis of the fatty acids and proteins found in the hemolymph [2], [3]. In our case, the impressive size reached by this tissue as a consequence of the acorn ingestion coincided with significant variations in the measured ecophysiological parameters. Thus, the hemolymph composition was altered by the diet, particularly in the fatty-acid type and content. All the fatty acids detected in the hemolymph of the cow dung-fed beetles (palmitic, palmitoleic, oleic and linoleic) were significantly increased two to three-fold in the hemolymph of acorn-fed beetles. In addition, the acorn diet caused the appearance of stearic and linolenic acids in the hemolymph. Both stearic and linolenic acids were undetectable in the cow dung diet. Insects utilize lipids efficiently for development, reproduction and flight, and the amount and composition of lipids in an insect vary considerably among the developmental stages and tissues and are influenced by several factors, including nutrition [26]. In insect physiology, polyunsaturated fatty acids have diverse roles in extreme temperature tolerance, reproduction and development [27], [28]. For example, in *Sarcophaga crassipalpis*, oleic acid promotes cell membrane fluidity at low temperature and also allows the cell membrane to maintain a liquid crystalline state if temperatures increase in the cell membrane [27].

Diet has not only an important effect on the fat body mass, but also on the cryoprotectant content of the hemolymph. In our study, the changes in the concentration of hemolymph fatty acids related with the acorn consumption seem to be associated to the temperature tolerance of *T. lusitanicus*. We found a significant reduction in the supercooling point (SCP) in the hemolymph from acorn fed dung-beetles. Besides the modification of the hemolymph SCP, the diet change also brought a change in the thermal hysteresis activity (THA) detected in the hemolymph. Interestingly, although the type and identity of these proteins is now being investigated in our laboratory, we suspect that this great difference in the protein content derived from the dietary shift is related to the changes in the biophysical characteristics of the hemolymph and the observed higher thermal tolerance and activity rates at lower temperatures. The effect of diet on thermal tolerance was also observed in other arthropods. In the Antarctic midge *Belgica antartica*, antifreeze and cryoprotectant modulation is dependent on the dietary carbon source [29], but this process does not seem to be universal [30]. In the case of *T. lusitanicus*, the consumption of acorns and the accompanying metabolic changes significantly increased the tolerance to lower temperatures. In fact, for an overwintering species such as *T. lusitanicus*, the capacity to increase the cold tolerance could be advantageous by enabling the beetle to survive hard frosts during winter, but also by extending its activity during the cold conditions in which other species remain inactive. In the locality at which this behavior was first observed [18], [19], *Thorectes lusitanicus* is the only dung beetle species that remains active in the field during winter according to our field experience. Our results on the activity test support this observation; those individuals feeding on acorns were more active at both temperatures considered but especially during cold conditions (10°C) maintaining 85.5% activity in contrast to individuals with a dung-exclusive diet which only 17.2% of individuals maintained activity (Mann-Whitney U test: U = 36, *P* = 0.001; for both temperature conditions) being also able to have a higher rate of survival to winter conditions. Preliminary laboratory observations suggest that a higher survival of *T. lusitanicus* individuals was obtained under a diet based on acorns compared to beetles under a dung-based diet (unpublished results).

In freeze-intolerant and overwintering species such as *T. lusitanicus*, the SCP and THA are two characteristics that increase during autumn to promote survival during the winter. In the case of *T. lusitanicus*, the modulation of the SCP and THA levels during the winter may be an important mechanism in preventing the formation of inoculative freezing (by contact with ice) during periods of frosts. In the distribution area of *T. lusitanicus*, snow and frost periods are scarce, although in some occasions the environmental temperature registered during winter can reach −5°C. However, a moderately chill-tolerant species such as *T. lusitanicus* can take advantage under natural situations when the SCP diminishes because the survival rate at low temperatures may drastically increase. In moderately chill-tolerant and chill susceptible species, changes in survival rates are related to SCP values [31] being the increase of SCP a frequent strategy to survive during coldest periods [32]. For example, in the case of the curculionid beetle *Rhynchaenus fagi*, although this species has an SCP of −25°C, less than 30% of individuals survive after 50 days at −15°C [33].

Acorn consumption seems to also affect the reproductive capacity of this species. Ovary weights, the number of oocytes per ovary and the mean weight of oocytes were significantly higher in the acorn-fed individuals (see Fig. 2C and Table 2) than in the beetles fed with cow dung, which also had a higher number of undeveloped ovaries. This higher ovarian development may be enhanced by the high content of fatty acids. Palmitic, oleic, and linoleic acids are the most abundant during the reproductive cycle of many insects [34]. Most insects have a nutritional requirement for polyunsaturated fatty acids, and many studies have pointed out that either linoleic or linolenic acids satisfy this nutritional need. The absence or deficiency of these compounds results in failures in metamorphosis, nymph growth, and the emergence of well-formed adults [35]. In the case of *T. lusitanicus*, a diet based on acorns not only provides high concentrations of fatty acids, but is also the unique source of the essential linolenic acid.

Is there any relationship between acorn density and population size or body size of *T. lusitanicus* under natural conditions? A review of the available data on the composition of dung beetle assemblages within the distribution area of *T. lusitanicus*, was shown in six published studies that collected this species [36], [37], [38], [39], [40], [41]. The total number of individuals collected in them oscillates between 3 and 241 (mean = 49 individuals) despite being annual studies. However, in the locality in which this surprising behavior has been discovered [18], the mean number of *T. lusitanicus* per trap in a 48-hours study was higher than the yearly mean of other sites. (51.4±5.1; n = 30; unpublished results). Moreover, variation in abundance between sampling plots was highly related to variation in acorn density (65% of the total variability; 2.5±0.8 (mean± se) individuals per m^2^ were collected directly in the soil [19], and 1,542 total individuals were collected). Preliminary field observations were carried out during November 2009 in four locations 20 km apart, and the locations characterized by their different tree cover values of *Quercus* species (approximately 90% of *Quercus suber*, 10% of *Q. suber*, 10% of *Q. rotundifolia* and 5% of *Q. rotundifolia*, respectively). The four locations had *T. lusitanicus* nest densities of 8.6±2.1, 3.2±0.8, 2.0±0.7, 1.2±0.6 (mean± se) nests/m^2^ (Kruskal-Wallis test: *T* = 11.89; *P*<0.01; *n* = 3 sites/location), respectively, preliminarly supporting the influence of the consumption acorns on the population size of *T. lusitanicus*. Furthermore, the mean body size of *T. lusitanicus* individuals also differed (Kruskal-Wallis test: *T* = 30.59; *P*<0.0001) between the surveyed localities. Individuals (*n* = 14, 10, 11 and 13, for each tree cover category) inhabiting the most dense forest of *Quercus* had a mean body size of 6.35±0.12 mm (mean± se), while those individuals belonging to the other tree cover categories had mean body sizes of 5.26±0.15, 5.18±0.09, and 4.79±0.12 mm, respectively. It is well established that both the nutritional quality and quantity of food determines the body size of emerged adult dung beetles [42]. Although more field studies are necessary to corroborate the former detected tendencies, the current evidence clearly suggest the existence of a positive association between the abundance of *Quercus* trees and the population and/or body sizes of *T. lusitanicus*.

Biochemical reactions and metabolic rates are highly dependent on temperature. Hence, fully or partly endothermic animals profit from the ability to heat themselves, because it enables them to adjust their temperature to enhance metabolic rates across a broad range of environmental conditions [43]. Although most animal groups (except birds and mammals) cannot completely regulate their body temperature, many have physiological, behavioral, and morphological strategies to adjust their body temperature, in response to thermal environmental changes [44]. The main physiological mechanism to elevate body temperature in dung beetles is the shivering of flight muscles [45]. However, in *Thorectes* wing muscles are atrophied because these species are apterous, having lost their capacity to fly. The apterism could have facilitated the evolutionary emergence of alternative strategies to elevate body metabolism through trophically mediated mechanisms. Alternatively, the acquisition of a new and energy-rich food source, that implies the increase of fat body, may increase the caloric energy by circulating metabolic substrates (lipids, proteins, polyalcohols, etc.), which are replenished by the fat body, and subsequently the tolerance to cold conditions, thus diminishing the selective pressure of the use of flight muscles to elevate body temperatures. A recent phylogenetically-based analysis showed that apterism evolved recurrently throughout Geotrupidae evolution (Cunha, Verdú, Lobo and Zardoya, in preparation). According to this phylogenetic hypothesis, *Thorectes baraudi* is the closest species to *T. lusitanicus*. However, *Mycotrupes lethroides* belongs to a relatively unrelated American lineage. The absence of phylogenetic propinquity in this diet-shift behavior suggest that apterism, a common condition of three species with this acorn diet-shift, could be the facilitation mechanism behind the evolutionary emergence of alternative diet-shift behavior in these dung beetles.

Most Geotrupinae species can be considered cool-temperature adapted species whose ancestral tribes are not strictly specialized in the consumption of herbivore dung. The ancestral shift in feeding habits from saprophagy to coprophagy in the Scarabaeoidea probably coincided with the appearance of large terrestrial herbivores, particularly grazing Mesozoic reptiles and subsequently mammals [46]. In general, an evolution towards coprophagy is evident in several Scarabaeoidea lineages. The diet of the most primitive geotrupids [47] is likely based on humus and fungi, whereas many Geotrupinae are basically coprophagous but possess modifications in the adult mouthparts that facilitate the consumption of more hard resources [48]. *Thorectes* species are characterized by the presence of mandibles with strongly developed scissorial areas and well-developed molar areas capable of triturating dry-pellet, an adaptation that suggest that these species are dung-fiber consumers and not dung-juice consumers [48]. Thus, although these species inevitably seem to require the presence of dung in order to build their nests and feed their larvae, their mouthparts are able to exploit trophic resources such as dry dung pellets and vegetable compounds (such as acorns). The wide range of trophic resources consumed by Geotrupidae dung beetles and the generally accepted saprophagous habits of the Scarabaeoidea ancestors [49], [50] suggest that the consumption of acorns constitutes a faculty that takes advantage of previous morphological adaptations.

This flightless Geotrupinae lineage would have evolved to reduce the loss of body water under arid conditions, as is common in other Coleoptera groups colonizing dry environments [51]. Subsequently, the loss of the flight capacity would have diminished the possibility of using an ephemeral resource such as dung, promoting the morphological adaptations directed to consume hard organic substances such as acorns, an evolutionary decision to maximize the net energy intake [14]. At the same time, this apterism would also have caused the isolation and diversification of this group. *Thorectes* (*sensu lato*) constitutes the most diversified Geotrupinae lineage in the Western Mediterranean region [52], with 36 endemic or narrowly distributed species (74% of the total, approximately) present in the Iberian Peninsula and North Africa, where the distribution seem to be highly conditioned by river-basin configurations [53]. In our opinion, both the capacity to consume hard resources and the apterism of *Thorectes lusitanicus*, *T. baraudi* and *Mycotrupes lethroides* are key characteristics that may explain the observed dietary shift and the astonishing relevance to thermal tolerance observed for *T. lusitanicus*. Mouthparts capable of crushing hard vegetable products would have facilitated the dietary shift towards a more energetically rich trophic resource such as acorns, which are able to provide the proteins and lipids necessary for enhancing cold resistance in the absence of the flight-muscle shiver mechanism.

In summary, the consumption of acorns by *T. lusitanicus* and the accompanying metabolic changes significantly increased the thermal tolerance of the species, and probably also the reproductive success of the species. Dietary acorn-selection by *T. lusitanicus* represents an unexpected behavior, which demonstrates that an apparently simple change in the diet can potentially drastically enhance the ecological capacity of an insect species, creating new life history possibilities from existing plasticity. Considering the large size of *T. lusitanicus* populations [18], more research is needed to examine the relevance of this dietary shift to the long term dynamic between the forest regeneration processes and the action of the large herbivore communities with which these *Thorectes* dung consumer species are associated. Furthermore, long-term additional evidences under natural conditions are necessary to establish a direct causal link between the acorn consumption and fitness or survival variables.

## Materials and Methods

### Study site and animals

Individuals of *Thorectes lusitanicus* were captured from the Los Alcornocales Natural Park in the Aljibe Mountains in southern Spain (36°31′54″N, 5°34′29″W) during the autumn (November-December 2006, 2007, 2008 and 2009), to coincide with the production of acorns and with the reproduction period of *T. lusitanicus*. Each year, in 48-h periods, we used pitfall traps baited with cow dung to capture live beetles. All of the individuals were maintained in plastic containers (60×40×40 cm) at 20°C until their arrival at the laboratory, where they were maintained at 10°C in a climate chamber (a temperature similar to the average one experienced in the field during autumn). The substrate was fallen leaves of *Quercus* to eliminate stress for the beetles.

We considered several methodological requirements to assure a common physiological state for all of the individuals, thus allowing the comparison of physiological measures between the diets: (a) we randomly selected only mature specimens according to cuticular deterioration of tibial in conjunction with the hardness of the cuticle employed in age determination of dung beetles [54], which permit the identification of individuals of approximately the same age; (b) a sex ratio of 1∶1 was maintained in each experiment; (c) to avoid the effect of diet and gut content on physiological measures, after a captive feeding period, all beetles were starved for a ten-day acclimation period prior to the SCP determination and extraction of hemolymph. The individuals submitted to different diets were separated in different rooms in order to avoid the presence of olfactory information on the two resources at the same time.

### Types of diet and acclimation temperatures

Two plastic containers (60×40×40 cm) with 100 individuals of *T. lusitanicus* (50 males and 50 females) were installed with a substrate of sterile dry vermiculite. These individuals were exclusively fed with randomly selected acorns. Two additional plastic containers with 100 individuals were fed cow dung exclusively. The four containers were placed in a climate chamber at 20°C, with 60% relative humidity (RH) with a photoperiod of 15∶9 (light∶dark). Individuals were supplied with fresh dung or acorns every four days to ensure that the food supply was not a limiting factor in the analysis. After 20 days, 50 animals from each container were randomly selected and placed into two new containers at 10°C, 60% RH, with a photoperiod of 15∶9 (light∶dark) without further feeding. After ten days in these conditions, the hemolymph was extracted and analyzed.

### Fat body mass and ovary development

Prior to the dissection, 20 females (n = 10 per treatment) were numbered and weighed. The fat body tissue and ovaries were dissected from ice-cold beetles into ice-cold insect Ringer's solution. For each individual, all the fat body tissue was removed and immediately placed on a dry paper to eliminate excess water prior to weighing. The same procedure was used for the ovary weight measurement. Also, the number of oocytes per ovary and their developmental status were noted. Finally, the fat body tissues and ovaries were stored at −85°C for later analyses.

### Extraction of hemolymph

For each treatment, samples of hemolymph were obtained in autumn-winter coinciding with the reproductive period of the species. Hemolymph collection was done by piercing the dorsal surface of the thorax with a pin and collecting the hemolymph with capillary tubes. Each individual extraction was transferred to a 1.5-ml Eppendorf vial, with a total of 10 individual extractions aggregated to form a single sample per vial. After the extraction of the hemolymph, samples and beetles were frozen and stored at –85°C.

### Protein analysis

The total protein content in the hemolymph was estimated using the Bradford method [55]. Briefly, the extracted hemolymph was centrifuged at 8000×g for 5 min to separate the particulate material. Then 20 µl-aliquots of the supernatant were diluted with water to a final volume of 100 or 1000 µl, depending on whether the samples came from excrement- or acorn-fed beetles, respectively. Aliquots of 100 µl each of the diluted samples were then mixed with 3 ml of Bradford reagent and left to stand at room temperature for 10 min. The absorbance at 595 nm of the mixture was measured in the context of the proper blank in a Shimadzu UV-VIS spectrophotometer UV-6001. The protein concentration was calculated from a calibration plot constructed under the same conditions as described above using bovine serum albumin (BSA) as the standard and taking into account the different dilution made with both samples.

### Glycerol analysis

The glycerol present in the hemolymph of the beetles was analyzed with the Glycerol Assay Kit from Megazyme®. This procedure is based in the phosphorylation of the glycerol present in the samples to L-glycerol-3-phosphate by a reaction catalyzed by glycerokinase, which yields adenosine-5′-diphosphate (ADP) as a by-product. The ADP formation is then coupled to the cleavage of pyruvate from phosphoenolpyruvate by pyruvate kinase. Finally, the pyruvate formed in this reaction is reduced to L-lactate by L-lactate-dehydrogenase with the concomitant formation of NAD^+^, which is photometrically assessed at 340 nm. The amount of NAD^+^ formed is stoichiometric with the amount of glycerol originally present in the samples. The concentration of glycerol in the samples was inferred from a Mega-Calc™ spreadsheet.

### Fatty acid analysis

To extract and analyze the fatty acids from the hemolymph, 30-µl aliquots of hemolymph were mixed with 440 µl chloroform: methanol (1∶2) and 100 µl of MethPrepII© methylating reagent. The mixture was maintained for 30 min in an ultrasound bath and then centrifuged to settle any particulate material. The fatty acid composition in the supernatant was analyzed by GC-MS.

### Supercooling point

**Figure 3:**
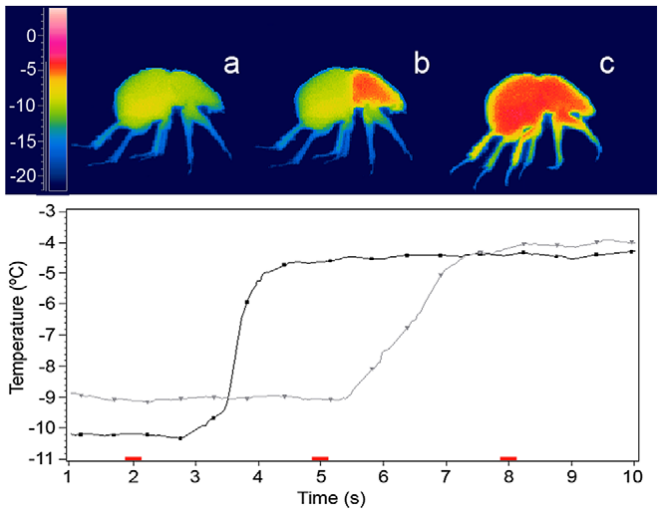
The supercooling process in *Thorectes lusitanicus*. (A) The prefreezing state when the supercooling point was measured; (B) the exothermic crystallization of the hemolymph contained in the thorax (see black curve below); and (C) the crystallization of the hemolymph contained in the abdomen (see grey curve below) and the rest of the body. The red marks indicate the instant that correspond with each thermography and temperature curves (the thoracic temperature in the black curve, and the abdominal temperature in the grey line).

The temperatures of cooling of the hemolymph below its freezing point without it becoming solid were measured with a FLIR Thermacam P620 thermal infrared camera with a resolution of 640×480 pixels and a microbolometer Focal Plane Array detector with a spectral range of 7.5–13 µm and a thermal sensitivity of ±0.06°C at 30°C. We used the ThermaCAM™ Researcher v 2.9 software [56] to record and analyse the thermal images obtained. The supercooling points were measured by determining the temperature at which the exothermic reaction of crystallization (latent heat of crystallization) occurs at the onset of freezing [57] (Fig. 3). In order to standardize all measures, we have selected the supercooling point obtained in the thorax (Fig. 3). We fixed the cooling rate at 0.5°C/min. The cooling rates were maintained using a MIR-153 programmable heated and cooled incubator (SANYO Electric Co., Ltd) with an accuracy of ±0.2°C. The supercooling point experiments were carried out with a hemolymph sample of at least ten individuals for each diet.

### Thermal hysteresis

**Figure 4:**
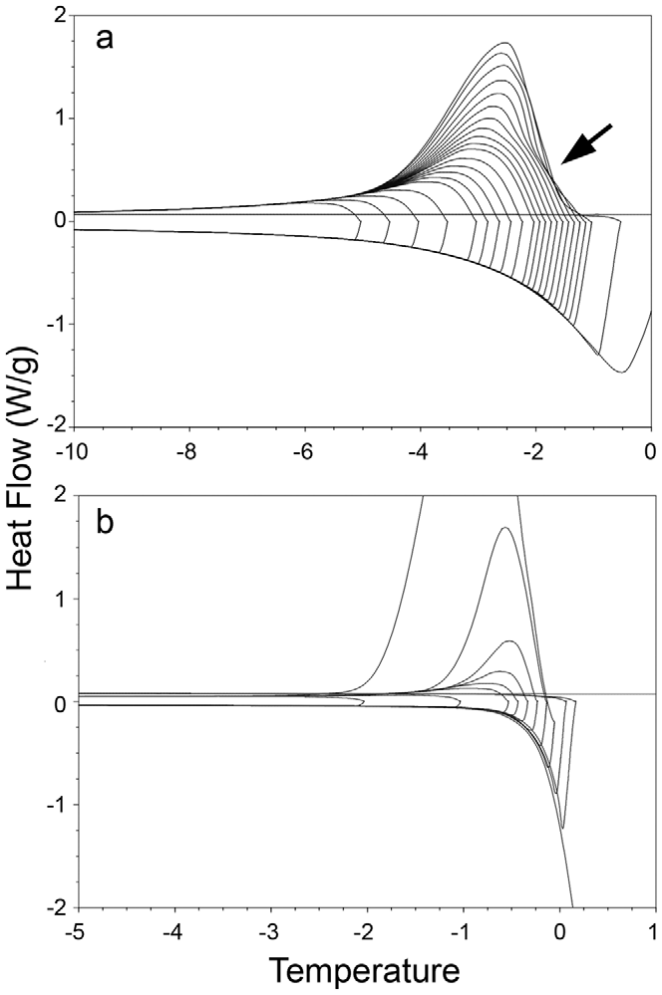
The thermal hysteresis activity (THA) of *Thorectes lusitanicus*. The differential scanning calorimetry curves of refreezing (rate of 0.1°C/min) of (A) partially melted hemolymph of *Thorectes lusitanicus*, and (B) partially melted bovine serum albumin (BSA; as control). No thermal hysteresis effect was observed in the curves with a lower heat flow (temperature ranges from –5 to –2°C), whereas THA was observed in temperature ranges oscillating from −2 to −1°C (see curves marked by an arrow). Frozen BSA was heated to partially melt at the different hold temperatures and then cooled to recrystallize. No thermal hysteresis effect was observed in this case.

These measurements were made using a differential scanning calorimeter (DSC; Q-100, TA Instruments). Each sample of hemolymph (1 µl) was sealed in an aluminum pan using an empty pan as control. The samples were weighed before the DSC run. Bovine serum albumin was used as a standard AFP-free solution to compare with samples containing AFP [58]. Following Hansen and Baust [59], the samples were cooled and warmed at 1°C/min, and both the crystallization and melting points were recorded. We used hemolymph without oil and varied some experimental parameters: the samples were then cooled and equilibrated, in this case, to −30°C (rate of 1°C/min), maintained for 5 min (isothermal time), warmed to successive partial melt temperatures between –10 and 0°C in 0.1°C increments, and held for 5 min at each increment to allow for the ice–protein interaction and system stabilization. Finally, the samples were then slowly cooled to −30°C (1°C/min), and the hold and onset of the freeze exothermal curve were recorded for each DSC cycle (Fig. 4). The onset was calculated as the start of the crystallization exothermal curve and was analyzed using the Universal Analysis 2000 v. 4.1D program (TA Instruments).

### Effect of diet on activity

In order to explore the diet effect on the activity of *T. lusitanicus*, twelve plastic containers (20×20×15 cm) with 20 individuals of *T. lusitanicus* (ten males and ten females) were installed with a substrate of vermiculite. Previously, six containers were supplied with acorns and six with cow dung during 20 days in 2008 and 2009. Three containers of each diet treatment were placed in separated climate chambers at 20°C or 10°C, 60% of relative humidity and a photoperiod of 15∶9 (light∶dark). Individuals were checked after four days, and the number of active individuals was counted. Individuals were only recorded as “active” if they exhibited normal behaviour, such as coordinated walking and extension of antennae. Null responses (inactive) correspond to immobile individuals in that as much the legs as the antennae remained folded to body during the experiment. This experiment was made during 2008 and 2009.

### Statistical analyses

Mann–Whitney non-parametric tests were used to examine the differences among the treatments using StatsDirect 2.5.7 [60].
